# The *LINC02381*/*let-7g-5p*/*THBS1* Signaling Axis Modulates Cellular Proliferative Activity in Osteosarcoma

**DOI:** 10.3390/cancers17193194

**Published:** 2025-09-30

**Authors:** Jing Wang, Shuming Hou, Ning Kong, Jiashi Cao, Xiangzhi Ni, Cheng Peng, Pei Yang, Kunzheng Wang

**Affiliations:** 1 Department of Orthopedics, Second Affiliated Hospital of Xi’an Jiaotong University, Xi’an 710000, China; wangj0415@163.com (J.W.); kongning19940621@stu.xjtu.edu.cn (N.K.); yangpei@xjtu.edu.cn (P.Y.); 2 Department of Orthopaedic Oncology, Spinal Tumor Center, Shanghai Changzheng Hospital, Naval Medical University, Shanghai 200000, China; 19802102212@163.com (S.H.); czyynxz@163.com (X.N.); pcwl777@163.com (C.P.); 3 Department of Orthopedics, No. 455 Hospital of Chinese People’s Liberation Army, Navy Medical University, Shanghai 200000, China; jiashicao1@126.com

**Keywords:** osteosarcoma, *LINC02381*, *let-7g-5p*, *THBS1*, viability, invasion, migration

## Abstract

**Objective:** This study aimed to elucidate the regulatory mechanisms of the long intergenic non-protein coding *RNA 02381* (*LINC02381*)/*microRNA-let-7g-5p* (*let-7g-5p*)/*thrombospondin 1* (*THBS1*) signaling axis in osteosarcoma (OS). **Methods:** The expression levels of *LINC02381*, *let-7g-5p*, and *THBS1* were quantified in OS and adjacent normal tissues via reverse transcription quantitative polymerase chain reaction. Their correlations with clinicopathological features were analyzed. Expression patterns were further validated in OS cell lines (143B, U-2OS, Saos-2, MNNG-HOS, MG-63) and normal osteoblast cell line hFOB1.19. The molecular interaction between *LINC02381* and *let-7g-5p* and the targeting relationship of *let-7g-5p* with *THBS1* were verified via dual-luciferase reporter and RNA pull-down assays. Functional effects were assessed using cell counting kit-8, colony formation, Transwell migration, and xenograft tumor models. **Results:** Compared to adjacent normal tissues, *LINC02381* and *THBS1* were upregulated in OS tissues (fold change > 3.0, *p* < 0.001), while *let-7g-5p* was downregulated (fold change ≈ 0.038, *p* < 0.001). Similar expression trends were observed in U-2OS cells. Knockdown of *LINC02381* or overexpression of *let-7g-5p* reduced cell proliferation, colony formation, migration, *THBS1* expression, and tumor volume (*p* < 0.001). These inhibitory effects were partially reversed by *let-7g-5p* inhibitors, restoring cell viability and migration by approximately 70%. Mechanistically, *LINC02381* functioned as a competing endogenous RNA (ceRNA), directly binding to *let-7g-5p* and mitigating its suppression of *THBS1*. **Conclusions:** *LINC02381* promotes OA progression by acting as a ceRNA for *let-7g-5p*, thereby upregulating *THBS1* expression. This signaling axis represents a potential therapeutic target for OS.

## Simple Summary

This study investigates the role of the *LINC02381/let-7g-5p/THBS1* signaling axis in the progression of osteosarcoma (OS). The expression levels of *LINC02381*, *let-7g-5p*, and *THBS1* were assessed in OS tissues and adjacent normal tissues using RT-qPCR, and their correlations with clinicopathological features were analyzed. Differential expression was further validated in OS cell lines and normal osteoblasts. Functional assays—including cell proliferation, colony formation, migration, and xenograft tumor growth—were conducted. The results indicated that *LINC02381* and *THBS1* were significantly upregulated, while *let-7g-5p* was downregulated in OS tissues and cells. Silencing *LINC02381* or overexpressing *let-7g-5p* suppressed cell proliferation, reduced colony formation and migration, decreased *THBS1* expression, and inhibited tumor growth in vivo. Mechanistically, *LINC02381* acts as a competing endogenous RNA (ceRNA) by binding to *let-7g-5p*, thereby preventing *let-7g-5p* from downregulating *THBS1*. These findings imply that *LINC02381* promotes OS progression via the *let-7g-5p/THBS1* axis and may serve as a promising therapeutic target.

## 1. Introduction

The existing evidence confirms that long non-coding RNAs (lncRNAs) can function as competitive endogenous RNAs (ceRNAs) by modulating microRNAs (miRNAs), thereby influencing tumor cell migration and proliferation [1]. Long intergenic non-protein coding *RNA 02381* (*LINC02381*), a member of the lincRNA family, has emerged a crucial regulator in oncogenesis. Recent studies have indicated that lincRNAs are actively involved in transcriptional reprogramming and play essential roles in malignant progression [1]. Aberrant *LINC02381* expression has been documented in various malignancies. For instance, it is significantly overexpressed in esophageal squamous cell carcinoma tissues compared to normal esophageal mucosa [2], and elevated levels have also been observed in glioma [3] and cervical carcinoma cells, where it exerts oncogenic functions contributing to tumor progression [1]. Notably, Xia Bian et al. reported that *LINC02381* knockdown suppresses osteosarcoma (OS) cell invasion, proliferation, and migration [4]. However, the precise molecular mechanisms through which *LINC02381* functions in OS remain largely undefined.

To explore its downstream regulatory network, we focused on miRNAs potentially interacting with *LINC02381*. Among them, *microRNA-let-7g-5p* (*let-7g-5p*) attracted our attention due to its established tumor-suppressive role. As a member of the let-7 family, *let-7g-5p* is known for its low expression in cancers such as nasopharyngeal carcinoma and cholangiocarcinoma [5,6]. Meanwhile, reduced *let-7g-5p* levels have been detected in osteoporotic fractures [7], although its role in OS remains poorly understood. Emerging evidence suggests that *let-7g-5p* may target oncogenes like *HMGA2* and *MAP3K1* [8,9], indicating its potential involvement in OS progression [10]. Bioinformatic predictions reveal potential binding sites between *let-7g-5p* and Thrombospondin-1 (*THBS1*), a stromal glycoprotein involved in tumor-associated processes such as cell–matrix interactions, extracellular matrix remodeling, and angiogenesis [11,12]. *THBS1* is frequently overexpressed in gastric and colorectal cancers and is related to poor prognosis [13,14]. Furthermore, elevated *THBS1* levels have been linked to osteoporosis [15], and its upregulation in OS has been correlated with aggressive tumor behavior and poor clinical outcomes [16,17,18]. Mechanistically, *THBS1* is believed to promote tumor progression by modulating the extracellular matrix, stimulating angiogenesis, and activating the *transforming growth factor beta* (*TGF-β*) signaling pathway [19]. However, the direct regulatory relationship between *THBS1* and *let-7g-5p* has not yet been established.

Based on these molecular insights, we propose that *LINC02381*, *let-7g-5p*, and *THBS1* may constitute a cascade regulatory network in OS. Supported by bioinformatic predictions and preliminary experimental data, we hypothesize that *LINC02381* modulates *let-7g-5p* activity via a ceRNA mechanism, thereby regulating *THBS1* expression and contributing to OS pathogenesis. Accordingly, this study aims to investigate the role of the *LINC02381*/*let-7g-5p*/*THBS1* axis in OS cell viability and tumor progression.

## 2. Materials and Methods

### 2.1. Ethics Statement

All experimental procedures were authorized by the Research Ethics Committee of Shanghai Changzheng Hospital, Navy Medical University in compliance with international ethical guidelines (approval number: 2017052, ethics approval data: 20170523). Written informed consent was obtained from all patients. All animal procedures were authorized by the Animal Ethics Review Board of the same institution (approval number: 2017122, ethics approval data: 20171225) and conducted following the NIH Guide for the Care and Use of Laboratory Animals as well as relevant local animal welfare regulations.

### 2.2. Experimental Subjects

A total of 71 patients with OS who underwent surgical resection at Shanghai Changzheng Hospital, Navy Medical University from July 2017 to July 2022 were included. Paired OS tumor tissues and paracancerous tissues were collected. Clinicopathological variables such as gender (male/female), age (≥20 vs. <20 years), tumor size (≥3 cm vs. <3 cm), TNM stage (I–II vs. III–IV), and lymph node metastasis (yes/no) were systematically recorded. Detailed information is presented in Table 1. All clinical definitions adhered to the *AJCC Cancer Staging Manual, 8th Edition*, and institutional diagnostic criteria established by the pathology and radiology departments. Data were independently reviewed and validated by two researchers. None of the enrolled subjects had received prior radiotherapy or chemotherapy. Collected tissues were labeled and cryopreserved at −70 °C for subsequent experiments.

To further investigate the correlation between molecular expression and treatment response, follow-up was conducted for all 71 patients who received postoperative adjuvant chemotherapy. According to RECIST criteria, patients were categorized into chemotherapy-sensitive (CR/PR) and chemotherapy-insensitive (SD/PD) groups based on radiological evaluations.

### 2.3. Cell Culture

Human OS cell lines (143B, U-2OS, Saos-2, MNNG-HOS, MG-63) and the human normal osteoblast cell line hFOB1.19 were purchased from the American Type Culture Collection (ATCC, Manassas, VA, USA). 143B and MNNG-HOS cells were cultured in RPMI-1640; Saos-2 in α-minimum Eagle’s medium (α-MEM); MG-63 in MEM; and U-2OS and hFOB1.19 in Dulbecco’s modified Eagle medium (DMEM), all supplied by Gibco (Grand Island, NY, USA). Media were supplemented with 10% fetal bovine serum (FBS) and 1% antibiotic–antimycotic solution (100 U/mL penicillin and 100 U/mL streptomycin). Cells were maintained in a 37 °C incubator with 5% CO_2_, with medium changes every two days. Subculturing was performed when cell confluence reached 80–90%.

Cells in the logarithmic growth phase after 2–3 stable passages were used for experiments. Total RNA was extracted using TRIzol reagent (Invitrogen, Carlsbad, CA, USA). RNA concentration and purity were assessed using a NanoDrop 2000 spectrophotometer (acceptable A260/A280 ratio: 1.8–2.1). The integrity of 28S/18S ribosomal RNA was confirmed by agarose gel electrophoresis to ensure RNA quality for downstream reverse transcription quantitative polymerase chain reaction (RT-qPCR) analysis. The mRNA expression levels of *LINC02381*, *let-7g-5p*, and *THBS1* were detected by RT-qPCR, and *THBS1* protein levels were assessed by Western blotting. Based on relative expression differences, U-2OS and Saos-2 cells—both exhibiting the greatest divergence from the normal osteoblast hFOB1.19—were selected for subsequent functional experiments.

### 2.4. Cell Grouping and Transfection

U-2OS and SaOS-2 cells in the logarithmic growth phase were seeded into 6-well plates at a density of 2 × 10^5^ cells per well. Transfection was initiated when cell confluence reached approximately 80%. Lipofectamine™ 2000 (Catalog No.: 11668-027, Invitrogen, Carlsbad, CA, USA) was used for transfection, strictly following the manufacturer’s protocol. Briefly, each oligonucleotide (final concentration: 50 nM; all synthesized by Shanghai GenePharma Co., Ltd., Shanghai, China) was diluted in 250 μL of serum-free DMEM and incubated at room temperature for 5 min. Separately, 5 μL of Lipofectamine™ 2000 was diluted in 250 μL of serum-free DMEM and incubated for another 5 min. The two solutions were then combined and allowed to form complexes at room temperature for 20 min before being added to the wells. Cells were incubated at 37 °C in a humidified incubator with 5% CO_2_ for 6 h. Afterward, the transfection medium was replaced with DMEM containing 10% FBS, and cells were cultured further for downstream applications.

Cells were divided into the following eight groups, with three replicates per group. Transfection efficiency was verified by RT-qPCR and/or Western blot prior to functional assays: short hairpin RNA (sh)-negative control (NC) group (transfected with the negative control of *LINC02381* knockdown vector), sh-*LINC02381* group (transfected with *LINC02381* knockdown vector), mimic-NC group (transfected with the negative control of *let-7g-5p* mimic), *let-7g-5p* mimic group (transfected with *let-7g-5p* mimic), sh-*LINC02381* + inhibitor-NC group (transfected with *sh-LINC02381* background with negative control miRNA inhibitor), sh-*LINC02381* + *let-7g-5p* inhibitor group (transfected with *sh-LINC02381* background with *let-7g-5p* inhibitor), sh-*LINC02381* + overexpression (oe)-NC group (co-transfected with the negative control of *THBS1* overexpression vector and *LINC02381* knockdown vector), and sh-*LINC02381* + oe-*THBS1* group (co-transfected with the *THBS1* overexpression plasmid and *LINC02381* knockdown vector).

### 2.5. Cell Counting Kit-8 (CCK-8) Assay

The proliferative capacity of U-2OS and SaOS-2 cells was assessed using a CCK-8 assay kit (Dojindo Molecular Technologies, Kumamoto, Japan). Cells from each group were seeded into 96-well plates at a density of 1 × 10^4^ cells per well, with three replicates per group and one blank well as an NC. Cells were incubated at 37 °C in a 5% CO_2_ atmosphere for 24, 48, and 72 h. At each time point, 10 μL of CCK-8 working solution was added directly to each well (without medium replacement), followed by a 4 h incubation under the same conditions. The optical density (OD) at 450 nm was measured using a microplate reader (Bio-Rad, Hercules, CA, USA). Cell proliferation curves were plotted accordingly. All procedures strictly adhered to the manufacturer’s instructions, and all experiments were independently repeated at least three times to ensure data reliability and reproducibility.

### 2.6. Colony Formation Assay

A plate colony formation assay was performed to evaluate the clonogenic potential of U-2OS and SaOS-2 cells in each experimental group. Cells in the logarithmic growth phase were seeded into 6 cm culture dishes at a density of 200 cells per dish (1 × 10^3^/mL, in 200 μL of cell suspension). The dishes were gently swirled to evenly distribute the cells and then incubated at 37 °C in a humidified incubator with 5% CO_2_ for 10 days.

Once visible colonies (each > 50 μm in diameter) were observed by the naked eye, the culture was terminated. The supernatant was carefully aspirated, and cells were washed twice with phosphate-buffered saline (PBS). Colonies were fixed with 96% ethanol for 10 min, stained with 1% crystal violet solution for 5 min, rinsed gently with tap water, and air-dried for approximately 1 h. The dishes were then inverted, and colonies were counted using a transparent grid film and an inverted microscope (Olympus Corporation, Tokyo, Japan). Each group included three replicates, and all experiments were repeated at least three times. The average number of colonies and colony formation rate (number of colonies/number of seeded cells × 100%) were recorded.

### 2.7. Transwell Assay

Transwell chambers with an 8.0 μm pore size (Corning, Corning, USA) were used to assess the migratory ability of U-2OS and SaOS-2 cells. At 24 h post-transfection, cells were digested, resuspended, and adjusted to a concentration of 1 × 10^5^/mL. A 200 μL serum-free cell suspension was added to the upper chamber, while 600 μL of complete medium containing 10% FBS was added to the lower chamber as a chemoattractant. The cells were incubated at 37 °C with 5% CO_2_ for 24 h.

After incubation, non-migrated cells on the upper surface of the membrane were carefully removed with a cotton swab. The inserts were then fixed with 4% paraformaldehyde for 30 min, washed twice with PBS, and stained with 0.5% crystal violet for 20 min. Following staining, the membranes were rinsed thoroughly and air-dried. Five randomly selected non-overlapping fields per chamber were imaged and counted under an inverted microscope (×200 magnification, Olympus, Tokyo, Japan). Each group included three parallel replicates, and the experiments were independently repeated at least three times. The mean number of migrated cells was used for statistical analysis.

### 2.8. RT-qPCR

Total RNA was extracted from tissues and cells using the TRIzol reagent (Invitrogen, Carlsbad, CA, USA) in accordance with the manufacturer’s instructions. RNA concentration and purity were assessed using a NanoDrop 2000 spectrophotometer (Thermo Fisher Scientific, Waltham, MA, USA), with an A260/A280 ratio between 1.8 and 2.1 considered acceptable. RNA integrity was confirmed by 1% agarose gel electrophoresis through visualization of intact 28S and 18S rRNA bands and evaluation of the 28S:18S ratio.

Complementary DNA (cDNA) was synthesized using the PrimeScript RT Master Mix Perfect Real Time kit (Takara, Kyoto, Japan). Each 10 μL reaction contained 500 ng of total RNA. The reaction conditions included denaturation at 70 °C for 5 min to eliminate secondary structures, cooling on ice for 3 min, reverse transcription at 37 °C for 60 min, and enzyme inactivation at 95 °C for 10 min. The resulting cDNA was stored at −20 °C for further analysis.

Quantitative real-time PCR was performed using SYBR Green chemistry. Each 20 μL reaction mixture consisted of 10 μL of 2× Taq MasterMix, 0.4 μL each of forward and reverse primers (5 μM), 1 μL of cDNA template, 0.4 μL of ROX Reference Dye, and nuclease-free water. Primer sequences were synthesized by Shanghai GenePharma Co., Ltd., Shanghai, China (Table 2).

The qPCR protocol was as follows: initial denaturation at 95 °C for 5 min, followed by 35 cycles of 94 °C for 45 s, 56 °C for 45 s, and 72 °C for 45 s, with a final extension at 72 °C for 5 min. Amplification specificity was verified using melting curve analysis. GAPDH was used as the internal control for *LINC02381* and *THBS1*, and U6 for *let-7g-5p*. Each sample was analyzed in triplicate. Relative gene expression was calculated using the 2^^ΔΔCt^ method. A no-template control was included in each run to monitor for contamination.

### 2.9. Western Blot Assay

Tissues and cells were lysed using RIPA lysis buffer (Cat# P0013C, Beyotime Biotechnology, Jiangsu, China), and total protein was collected by centrifugation at 12,000× *g* for 15 min at 4 °C. Protein concentrations were determined with a BCA protein assay kit (Beyotime, Jiangsu, China). For each sample, 30 μg of protein in 20 μL loading buffer was separated on 15% SDS-PAGE gels under the following conditions: pre-electrophoresis at 80 V for 40 min, followed by separation at 120 V for 70 min.

Proteins were transferred onto PVDF membranes (Millipore, Burlington, MA, USA) using a Bio-Rad (Hercules, CA, USA) electrotransfer system at 100 V for 90 min. The membranes were blocked in 5% non-fat milk (prepared in TBST buffer) at room temperature for 1 h, then incubated overnight at 4 °C with the following primary antibodies: anti-THBS1 (ab267388, Abcam, Cambridge, UK; 1:1000) and anti-GAPDH (sc-32233, Santa Cruz Biotechnology, Dallas, TX, USA; 1:1000). After three 10 min washes with TBST, membranes were incubated for 1 h at room temperature with HRP-conjugated secondary antibodies (Goat anti-Rabbit IgG-HRP, Bio-Rad, Hercules, CA, USA; 1:5000).

Protein bands were visualized using an enhanced chemiluminescence (ECL) kit (Millipore, Burlington, MA, USA) and exposed to X-ray film (Kodak, Rochester, NY, USA). Band intensities were analyzed using ImageJ software (version 1.53k, NIH, Bethesda, MD, USA), and target protein levels were normalized to GAPDH.

### 2.10. Dual Luciferase Reporter Assay

The binding sites between *LINC02381* and *let-7g-5p* were predicted using the Starbase database (https://starbase.sysu.edu.cn/agoClipRNA.php). The wild-type (Wt) sequence of *LINC02381* containing the predicted binding site for *let-7g-5p* was synthesized and cloned into the psiCHECK2 luciferase reporter vector (Promega, WI, USA), generating the LINC02381-Wt construct. A mutant version (LINC02381-Mut) was created using the QuikChange XL Site-Directed Mutagenesis Kit (Agilent Technologies, Santa Clara, CA, USA). U-2OS and SaOS-2 cells at 70–80% confluence were co-transfected with either *let-7g-5p* mimic or mimic negative control (mimic-NC) along with LINC02381-Wt or LINC02381-Mut using Lipofectamine^TM^ 2000 (Invitrogen, Carlsbad, CA, USA). After 48 h, luciferase activity was measured using a Dual-Luciferase^®^ Reporter Assay System (Promega, Madison, WI, USA).

The same method was employed to verify the targeting relationship between *let-7g-5p* and the 3′UTR of *THBS1*.

### 2.11. RNA-Pull Down Assay

To validate the direct interaction between *let-7g-5p* and *LINC02381*, biotin-labeled wild-type (Wt) and mutant (Mut) probes of *let-7g-5p* were synthesized by Shanghai GenePharma Co., Ltd. (Shanghai, China) U-2OS and SaOS-2 cells (2 × 10^5^ cells/well in 6-well plates) were transfected with these probes using Lipofectamine™ 2000. After 48 h of incubation, cells were lysed with RNA pull-down lysis buffer (Ambion, Austin, TX, USA) on ice for 10 min. Lysates were centrifuged, and supernatants were incubated with streptavidin-coated magnetic beads (M-280 Dynabeads, Sigma-Aldrich, Saint Louis, MO, USA) pre-blocked with 0.1 mg/mL yeast tRNA and 0.1 mg/mL RNase-free BSA for 30 min. Bead-lysate mixtures were rotated at 4 °C for 3 h.

The beads were then washed sequentially: twice with cold lysis buffer, three times with low-salt buffer (50 mM NaCl), and once with high-salt buffer (500 mM NaCl) to eliminate nonspecific binding. The unlabeled Mut probe served as the negative control. RNA bound to the beads was extracted using TRIzol (Invitrogen, Carlsbad, CA, USA), reverse transcribed (PrimeScript RT Master Mix, Takara, Kyoto, Japan), and LINC02381 enrichment was quantified via qPCR. Each group was tested in triplicate, and experiments were independently repeated three times.

### 2.12. Tumor Xenografts in Nude Mice

Male BALB/c nude mice (18–20 g and 4–5 weeks old) were maintained under specific pathogen-free conditions. U-2OS and SaOS-2 cells stably transfected with sh-NC, sh-*LINC02381*, mimic-NC, or *let-7g-5p* mimic were subcutaneously injected into the flanks (2 × 10^6^ cells per mouse, with 7 nude mice in each group). Tumor volumes were measured every 7 days and calculated using the formula: (length × width^2^)/2. On day 28, mice were euthanized, tumors were harvested, excised, and weighed. Power analysis using G*Power v3.1 software (α = 0.05, effect size f = 0.8) confirmed that 7 mice per group would achieve >85% power for detecting differences in tumor volume and weight via one-way analysis of variance (ANOVA).

### 2.13. Statistical Analysis

Data analysis was carried out using SPSS v21.0 (IBM, Armonk, NY, USA). Measurement data following a normal distribution were presented as mean ± standard deviation. Differences between two groups were analyzed using the independent samples *t*-test, while multiple-group comparisons were performed via one-way ANOVA with Tukey’s post-hoc test. Fisher’s exact test was used to assess the relationships between *LINC02381*/*let-7g-5p* expression and clinicopathological characteristics. Quality control measures included assessment of data normality using the Shapiro–Wilk test, homogeneity of variance via the Levene test, and outlier detection using the Grubbs test (α = 0.05). Abnormal results due to technical error were excluded and experiments repeated. Animal grouping was randomized, and both tumor measurements and endpoint evaluations were conducted in a blinded manner. Meanwhile, in vitro experiments such as cell proliferation, migration, and dual-luciferase assays were also implemented and interpreted independently to minimize bias. A *p*-value below 0.05 was considered statistically significant.

## 3. Results

*LINC02381* and *let-7g-5p* expression are linked to lymph node metastasis (LNM) and tumor node metastasis (TNM) staging of patients with OS

**Figure 1:**
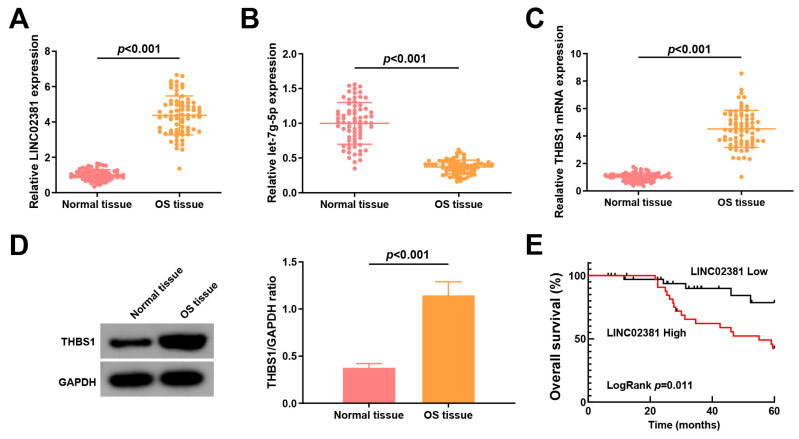
*LINC02381* and *let-7g-5p* expression are linked to TNM staging and LNM of patients with OS. (**A**) LINC02381 expression in OS tissues; (**B**) *let-7g-5p* expression in OS tissues; (**C**,**D**). *THBS1* mRNA and protein expression in OS tissues; (**E**) Kaplan-Meier survival analysis.

Initially, *LINC02381*, *let-7g-5p*, and *THBS1* expression levels in adjacent normal tissues and OS tissues were measured. *LINC02381* was upregulated in OS tissues (fold change ≈ 3.77, *p* < 0.001, Figure 1A), while *let-7g-5p* was downregulated (fold change ≈ 0.38, *p* ≤ 0.001, Figure 1B), suggesting a potential regulatory interaction.

Furthermore, *THBS1*, a predicted target of *let-7g-5p*, was significantly upregulated at both the mRNA (fold change ≈ 2.77, *p* < 0.001, Figure 1C) and protein level (fold change ≈ 3.03, *p* < 0.001, Figure 1D) in OS tissues. Kaplan–Meier survival analysis revealed that high *LINC02381* expression was associated with shorter overall survival (Log-rank *p* = 0.0448, Figure 1E).

Patients with OS were stratified into low- and high-expression groups based on the mean levels of *LINC02381* or *let-7g-5p*. High *LINC02381* or low *let-7g-5p* expression was significantly associated with advanced TNM stage (III–IV) and the presence of LNM (all *p* < 0.05), whereas no significant correlation was observed with gender, tumor size, or age (all *p* > 0.05) (Table 1).

To assess the relationship between molecular expression and therapeutic response, follow-up analysis was performed in 71 OS patients who underwent postoperative adjuvant chemotherapy. Based on RECIST criteria, patients were divided into chemotherapy-sensitive (CR/PR) and chemotherapy-insensitive (SD/PD) groups. Notably, the proportion of chemotherapy-insensitive patients was higher in the high *LINC02381* expression group (*p* = 0.008), while elevated *let-7g-5p* expression was associated with improved chemotherapy response (Table 3).

### 3.1. LINC02381 and THBS1 Are Upregulated and let-7g-5p Is Downregulated in OS Cell Lines

**Figure 2:**
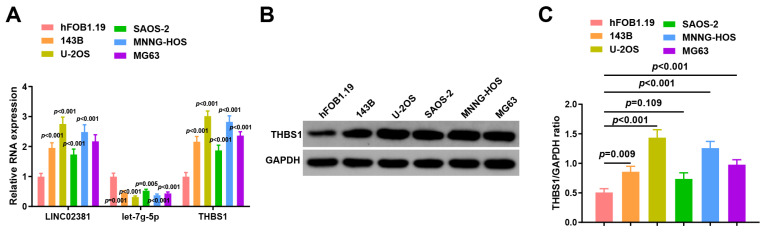
*LINC02381* and *THBS1* are increased and *let-7g-5p* is decreased in OS cell lines. (**A**) *LINC02381*, *let-7g-5p*, and *THBS1* expression of each cell line; (**B**,**C**) *THBS1* protein expression of each cell line.

To further validate these findings, we assessed the expression of *LINC02381*, *let-7g-5p*, and *THBS1* in human normal osteoblasts (hFOB1.19) and five OS cell lines (143B, U-2OS, SaOS-2, MNNG-HOS, and MG63) using RT-qPCR and Western blot. Compared to hFOB1.19, *LINC02381* was significantly upregulated in all OS cell lines (*p* < 0.001, *p* < 0.001, *p* < 0.001, *p* = 0.001, *p* < 0.001), while *let-7g-5p* was consistently downregulated (*p* < 0.001, *p* < 0.001, *p* = 0.005, *p* < 0.001, *p* < 0.001). Correspondingly, *THBS1* expression was increased across all OS cell lines (*p* < 0.001, *p* < 0.001, *p* < 0.001, *p* < 0.001, *p* < 0.001) (Figure 2A).

At the protein level, the expression of *THBS1* was markedly higher in U-2OS cells (*p* = 0.009), 143B, SAOS-2, and MNNG-HOS cells (*p* < 0.001) (Figure 2B,C).

Based on these results, U-2OS and SAOS-2 cells with a high expression of *LINC02381* and *THBS1* and a low expression of *let-7g-5p* were finally selected for subsequent functional experiments.

### 3.2. LINC02381 Regulates THBS1 Expression by Targeting let-7g-5p

Previous studies have demonstrated that *LINC02381* regulates the process of cancer cells by interacting with miRNAs. For example, it promotes the viability and migration of cervical cancer cells by targeting *miR-133b* [1]. Based on this evidence, we speculated that *LINC02381* may exert regulatory effects in OS by regulating *let-7g-5p*.

**Figure 3:**
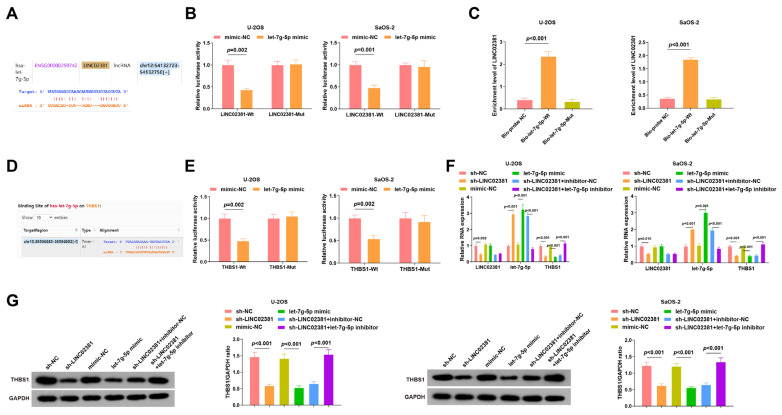
*LINC02381* regulates *THBS1* expression by targeting *let-7g-5p*. (**A**) Binding sites of *LINC02381* and *let-7g-5p* predicted by the bioinformatics website; (**B**) regulatory relationship between *LINC02381* and *let-7g-5p* in U2OS and SaOS-2 cells detected by dual luciferase reporter gene assay; (**C**) regulatory relationship between *LINC02381* and *let-7g-5p* in U2OS and SaOS-2 cells verified by RNA-pull down assay; (**D**) binding sites of *let-7g-5p* and *THBS1* predicted by the bioinformatics website; (**E**) regulatory relationship between *let-7g-5p* and *THBS1* in U2OS and SaOS-2 cells detected by dual luciferase reporter gene assay; (**F**,**G**) *LINC02381*, *let-7g-5p*, and *THBS1* expression in U2OS and SaOS-2 cells detected by qPCR and WB.

Bioinformatic analysis predicted potential binding sites between *LINC02381* and *let-7g-5p* (Figure 3A). Dual-luciferase reporter assays in U-2OS and SaOS-2 cells confirmed this interaction: co-transfection with LINC02381-Wt and let-7g-5p mimic significantly reduced luciferase activity compared to the mimic-NC group (U-2OS: *p* = 0.002; SaOS-2: *p* < 0.001), whereas no significant change was observed in the LINC02381-Mut group (*p* > 0.05) (Figure 3B). These findings indicate specific binding between *let-7g-5p* and *LINC02381*. The interaction was further validated via RNA pull-down assays, which showed significant enrichment of *LINC02381* by the biotin-labeled *let-7g-5p* probe (U-2OS: *p* < 0.001; SaOS-2: *p* < 0.001) (Figure 3C).

Target prediction tools identified *THBS1* as a putative downstream target of *let-7g-5p* (Figure 3D). This relationship was confirmed by dual-luciferase assays: co-transfection of THBS1-Wt with the let-7g-5p mimic led to reduced luciferase activity (U-2OS: *p* = 0.002; SaOS-2: *p* = 0.002), whereas THBS1-Mut showed no significant change (*p* > 0.05), indicating that *THBS1* is a direct target of *let-7g-5p* (Figure 3E).

Subsequent RT-qPCR and Western blot analyses demonstrated that either knockdown of *LINC02381* or overexpression of *let-7g-5p* significantly downregulated THBS1 expression (all *p* < 0.01, Figure 3F,G). In addition, sh-*LINC02381* elevated *let-7g-5p* levels, an effect that was reversed by co-transfection with the *let-7g-5p* inhibitor (*p* < 0.01), further proving that *LINC02381* can promote the expression of *THBS1* by acting as a molecular sponge for *let-7g-5p*.

### 3.3. LINC02381 Modulates Cell Proliferation and Migration via let-7g-5p

**Figure 4:**
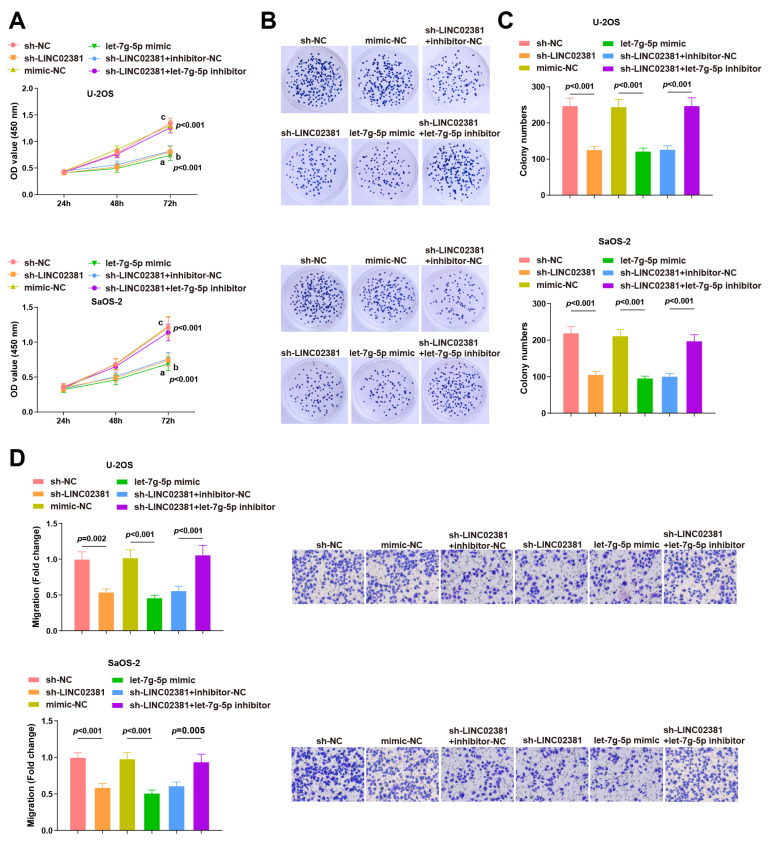
*LINC02381* modulates cell proliferation and migration through the regulation of *let-7g-5p*. (**A**) Proliferation of U2OS and SaOS-2 cells detected by CCK-8 assay; (**B**,**C**) colony formation ability of U2OS and SaOS-2 cells detected by colony formation assay; (**D**) migration ability of U2OS and SaOS-2 cells detected by Transwell assay.

To investigate the functional consequences of the *LINC02381/let-7g-5p* axis in OS, we performed CCK-8 and colony formation assays. In both U-2OS and SaOS-2 cells, knockdown of *LINC02381* or overexpression of *let-7g-5p* significantly inhibited cell proliferation (U-2OS: *p* < 0.001; SaOS-2: *p* < 0.001) and reduced colony formation (U-2OS: *p* < 0.001; SaOS-2: *p* = 0.001). Notably, co-treatment with the *let-7g-5p* inhibitor reversed the suppressive effects of sh-LINC02381 on proliferation and colony formation (U-2OS: *p* < 0.001; SaOS-2: *p* = 0.001) (Figure 4A–C).

Cell migration capacity, assessed using Transwell migration assays, revealed similar trends. Silencing *LINC02381* or overexpressing *let-7g-5p* markedly inhibited migration in both cell lines (U-2OS: *p* = 0.002 and *p* < 0.001; SaOS-2: *p* < 0.001 and *p* = 0.001), while co-transfection with the *let-7g-5p* inhibitor rescued this migratory defect (U-2OS: *p* < 0.001; SaOS-2: *p* = 0.005) (Figure 4D).

Collectively, these findings suggest that *LINC02381* regulates *THBS1* by targeting *let-7g-5p*, thereby affecting the proliferation and migration ability of OS cells.

### 3.4. THBS1 Reverses the Effects of Both LINC02381 and let-7g-5p in OS Cells

To clarify whether *THBS1* can mediate the regulatory effects of the *LINC02381*/*let-7g-5p* axis on the biological functions of OS cells, rescue experiments were conducted in U-2OS and SaOS-2 cells by co-transfecting sh-*LINC02381* with either oe-NC or oe-*THBS1*.

**Figure 5:**
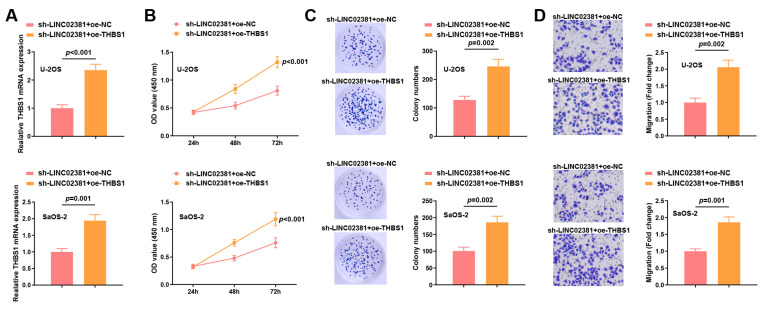
*THBS1* reverses the effects of both *LINC02381* and *let-7g-5p* in OS cells. (**A**) *THBS1* mRNA expression in U2OS and SaOS-2 cells detected by RT-qPCR; (**B**) viability of U2OS and SaOS-2 cells detected by CCK-8 assay; (**C**) colony formation ability of U2OS and SaOS-2 cells detected by colony formation assay; (**D**) migration ability of U2OS and SaOS-2 cells detected by Transwell assay.

qPCR results showed that compared with the sh-*LINC02381* + oe-NC group, the expression of *THBS1* in the sh-*LINC02381* + oe-*THBS1* group was increased (U-2OS: *p* < 0.001; SaOS-2: *p* = 0.001) (Figure 5A).

Functional assays showed that *THBS1* overexpression significantly reversed the inhibitory effects of *LINC02381* silencing. CCK-8 and colony formation assays revealed restored proliferative capacity (U-2OS: *p* < 0.001; SaOS-2: *p* < 0.001) (U-2OS: *p* = 0.002; SaOS-2: *p* = 0.002) (Figure 5B,C). Transwell migration assays further demonstrated that *THBS1* overexpression significantly rescued the migration ability suppressed by *LINC02381* knockdown (U-2OS: *p* = 0.002; SaOS-2: *p* = 0.001) (Figure 5D).

These findings indicate that *THBS1*, as a downstream effector, plays a crucial role in mediating the pro-tumorigenic functions of the *LINC02381/let-7g-5p* axis in OS cells.

### 3.5. Xenograft Tumor Experiment in Nude Mice

To further validate the role of *LINC02381* and *let-7g-5p* in vivo, U-2OS cells from the sh-NC, sh-*LINC02381*, agomir-NC, and *let-7g-5p* agomir groups (2 × 10^6^ cells/mouse) were subcutaneously injected into the flanks of 4–5-week-old male nude mice. Tumor dimensions were measured every 7 days to calculate volume and plot growth curves.

**Figure 6:**
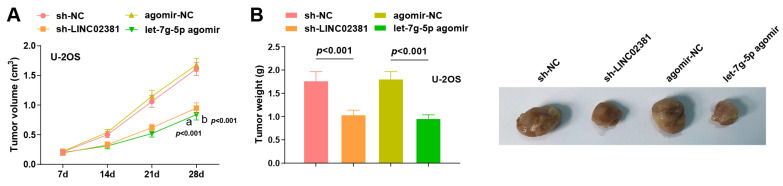
Xenograft tumor experiment in nude mice. (**A**) Tumor growth curve of nude mice after tumor xenografts with U2OS cells; (**B**) tumor weight of nude mice and illustration after tumor xenografts with U2OS cells. a *p* < 0.05 compared with the sh-NC group; b *p* < 0.05 compared with the mimic-NC group.

Tumors began to visibly grow by day 7 post-inoculation in all groups. From day 14 onward, tumor volumes in both the sh-*LINC02381* group and the *let-7g-5p* agomir group were notably smaller than their respective controls. On day 28, tumor volume was significantly reduced in the sh-*LINC02381* group compared to the sh-NC group (*p* < 0.001), and similarly in the *let-7g-5p* agomir group versus the agomir-NC group (*p* < 0.001) (Figure 6A).

Following euthanasia on day 28, tumor weights were recorded. Both *LINC02381* knockdown and *let-7g-5p* overexpression significantly reduced tumor weight compared to their controls (*p* < 0.001) (Figure 6B).

Collectively, these in vivo findings confirm that both *LINC02381* silencing and *let-7g-5p* upregulation effectively inhibit OS tumor growth, reinforcing their critical roles in OS progression and potential as therapeutic target.

## 4. Discussion

OS is a prevalent primary malignant bone tumor, predominantly affecting adolescents [20]. In this study, we demonstrated that *LINC02381* is significantly involved in the pathogenesis of OS through modulation of the *let-7g-5p*/*THBS1* axis. Mechanistically, *LINC02381* acts as a molecular sponge for *let-7g-5p* that sequesters *THBS1* thereby relieving its suppressive effect on *THBS1*, and consequently enhancing OS cell proliferation, migration, and invasion in vitro and in vivo.

Our study revealed upregulated *LINC02381* and *THBS1* expression, alongside downregulated *let-7g-5p* levels in OS tissues compared to normal controls. Furthermore, *LINC02381* and *let-7g-5p* expression levels correlated with TNM staging and LNM in OS patients, indicating their clinical relevance in OS progression. These findings are consistent with prior reports showing that *LINC02381* is aberrantly expressed in various malignancies [2,3], with its overexpression linked to advanced disease stages and poor outcomes [4]. Additionally, *let-7g-5p* downregulation has been observed in multiple cancers, including cholangiocarcinoma and glioblastoma [6,9], and its re-expression can inhibit cancer cell proliferation, migration, and invasion [10]. The overexpression of *THBS1* in OS tissues, as previously reported [17], further reinforces its oncogenic role.

Through dual-luciferase reporter and RNA pull-down assays, we validated the direct interaction between *let-7g-5p* and the 3′UTRs of both *LINC02381* and *THBS1*, confirming a ceRNA regulatory mechanism. Functional assays demonstrated that *INC02381* could regulate *THBS1* expression by targeting *let-7g-5p*, thereby influencing OS cell proliferation and migration. Importantly, overexpression of *THBS1* mitigated the inhibitory effects of *LINC02381* knockdown, highlighting *THBS1* as a critical downstream effector of this axis. These findings align with existing evidence that lncRNAs can function as ceRNAs in tumorigenesis. For instance, *LINC02381* has been shown to promote cervical cancer progression through sponging *miR-133b* [1], and to upregulate *CTNNB1* in endometriosis via *miR-27b-3p* [21]. In OS specifically, *LINC02381* was reported to promote malignancy by downregulating *CDCA4* via sponging *miR-503-5p* [4]. Together, these data suggest that *LINC02381* may operate through multiple ceRNA pathways, possibly exhibiting functional redundancy across different oncogenic contexts.

Notably, *THBS1* is a multifunctional matricellular protein that relies on downstream signaling pathways for its tumor-promoting activities. The literature indicates that *THBS1* regulates cell migration by binding to integrin receptors, as well as interacting with *CD47* and *CD36*, which modulate integrin-mediated signaling [22]. *THBS1* is also known to activate latent TGF-β1, contributing to bone metastasis in prostate cancer by facilitating TGF-β signaling [23]. These mechanisms are highly relevant to OS, particularly in the context of lung metastasis, as integrin β3 has been implicated in metastatic OS cell lines, and its silencing significantly impairs invasion and metastasis [24]. Moreover, *TGF-β* pathway overactivation has been associated with chemoresistance in OS [25]. Based on these insights, future research should incorporate phosphoproteomic profiling to assess downstream signaling alterations following *THBS1* overexpression and apply pharmacological inhibitors of integrin or *TGF-β* receptors to further dissect *THBS1*-mediated pathways.

## 5. Conclusions

**Figure 7:**
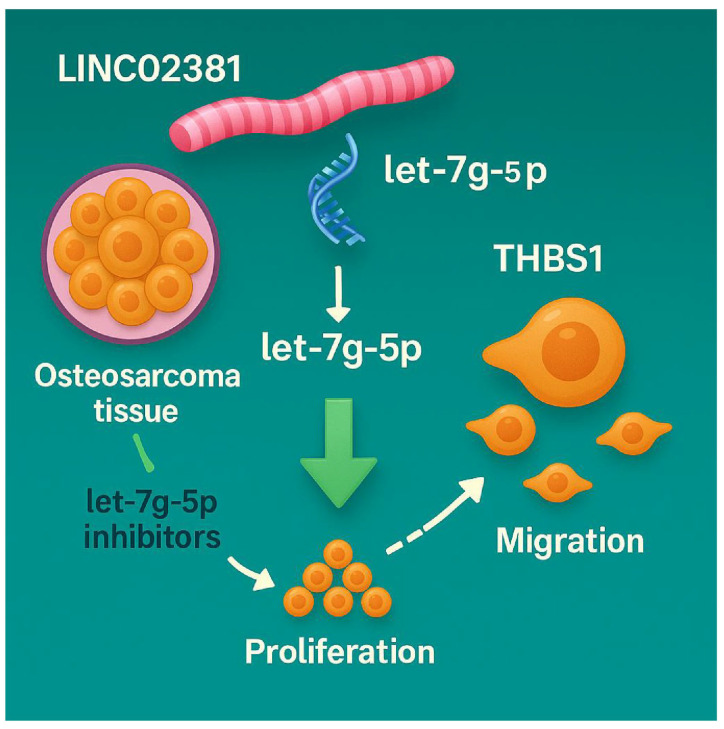
Diagram of the mechanism by which the *LINC02381/let-7g-5p/THBS1* axis promotes osteosarcoma progression.

In summary, this study confirms that *LINC02381* promotes OS progression by functioning as a molecular sponge for *let-7g-5p*, thereby upregulating *THBS1* expression and promoting OS cell proliferation, migration, and invasion both in vitro and in vivo (Figure 7). This axis represents a novel regulatory pathway in OS and offers potential targets for future therapeutic strategies. However, several limitations must be acknowledged. Specifically, the downstream signaling pathways of *THBS1* remain incompletely characterized, and clinical translation of RNA-based therapeutics faces significant challenges, including oligonucleotide instability, tissue-specific delivery, and off-target effects. Therefore, future studies should incorporate advanced delivery systems and mechanistic pathway analyses to enhance the feasibility of targeted interventions in OS.

## Figures and Tables

**Table 1:** The correlation between LINC02381 and let-7g-5p expression and clinicopathological characteristics of patients with OS.

Clinicopathological Data	*n*	*LINC02381* Expression		*p*	*let-7g-5p* Expression		*p*
		Low Expression Group (*n* = 35)	High Expression Group (*n* = 36)		Low Expression Group (*n* = 35)	High Expression Group (*n* = 36)	
Age (years old)							
≥20	42	24	18	0.149	23	19	0.337
<20	29	11	18		12	17	
Gender							
Male	38	18	20	0.814	16	22	0.238
Female	33	17	16		19	14	
TNM staging							
I–II	54	33	21	0.001	22	32	0.013 *
III–IV	17	2	15		13	4	
Tumor size							
≥3 cm	30	17	13	0.341	19	11	0.056
<3 cm	41	18	23		16	25	
Lymph node metastasis							
Yes	51	19	32	0.002	34	17	0.000 *
No	20	16	4		1	19	

Note: the data were enumeration data, using Fisher’s test; TNM, tumor node metastasis. * *p* < 0.05 indicates a statistically significant difference.

**Table 2:** Primer sequence for genes in this study.

Gene	Primer Sequence (5′-3′)
*LINC02381*	Forward: 5′-CCCTGCCCATAAGCTACTCA-3′
	Reverse: 5′-AACTTTGACCCCCAAATGCC-3′
*let-7g-5p*	Forward: 5′-GGGTGAGGTAGTAGTTTGT-3′
	Reverse: 5′-CAGTGCGTGTCGTGGAGT-3′
*TBHS1*	Forward: 5′-GCTGGAAATGTGGTGCTTGTCC-3′
	Reverse: 5′-CTCCATTGTGGTTGAAGCAGGC-3′
*U6*	Forward: 5′-ATTGGAACGATACAGAGAAGATT-3′
	Reverse: 5′-GGAACGCTTCACGAATTTG-3′
*GAPDH*	Forward: 5′-AACGTGTCAGTGGTGGACCTG-3′
	Reverse: 5′-AGTGGGTGTCGCTGTTGAAGT-3′

Note: GAPDH, glyceraldehyde-3-phosphate dehydrogenase.

**Table 3:** Relationship between molecular expression and chemotherapy response.

Molecular Group	Chemotherapy-Sensitive (CR/PR)	Chemotherapy-Insensitive (SD/PD)	*p*-Value
*LINC02381* low-expression (*n* = 35)	25	10	
*LINC02381* high-expression (*n* = 36)	15	21	0.008
*let-7g-5p* low-expression (*n* = 35)	14	21	
*let-7g-5p* high-expression (*n* = 36)	26	10	0.015

## Data Availability

The experimental data used to support the findings of this study are available from the corresponding author upon request.
